# Normalizing the Abnormal: Do Antipsychotic Drugs Push the Cortex Into an Unsustainable Metabolic Envelope?

**DOI:** 10.1093/schbul/sbz119

**Published:** 2019-11-22

**Authors:** Federico E Turkheimer, Pierluigi Selvaggi, Mitul A Mehta, Mattia Veronese, Fernando Zelaya, Paola Dazzan, Anthony C Vernon

**Affiliations:** 1 Department of Neuroimaging, Institute of Psychiatry, Psychology and Neuroscience, King’s College London, London, UK; 2 MRC Centre for Neurodevelopmental Disorders, King’s College London, London, UK; 3 Department of Psychosis Studies, Institute of Psychiatry, Psychology and Neuroscience, King’s College London, London, UK; 4 Department of Basic and Clinical Neuroscience, Institute of Psychiatry, Psychology and Neuroscience, King’s College London, London, UK

**Keywords:** antipsychotics, glucose metabolism, oxidative metabolism, long-term effects, symptom normalization, metabolic normalization

## Abstract

The use of antipsychotic medication to manage psychosis, principally in those with a diagnosis of schizophrenia or bipolar disorder, is well established. Antipsychotics are effective in normalizing positive symptoms of psychosis in the short term (delusions, hallucinations and disordered thought). Their long-term use is, however, associated with side effects, including several types of movement (extrapyramidal syndrome, dyskinesia, akathisia), metabolic and cardiac disorders. Furthermore, higher lifetime antipsychotic dose-years may be associated with poorer cognitive performance and blunted affect, although the mechanisms driving the latter associations are not well understood. In this article, we propose a novel model of the long-term effects of antipsychotic administration focusing on the changes in brain metabolic homeostasis induced by the medication. We propose here that the brain metabolic normalization, that occurs in parallel to the normalization of psychotic symptoms following antipsychotic treatment, may not ultimately be sustainable by the cerebral tissue of some patients; these patients may be characterized by already reduced oxidative metabolic capacity and this may push the brain into an unsustainable metabolic envelope resulting in tissue remodeling. To support this perspective, we will review the existing data on the brain metabolic trajectories of patients with a diagnosis of schizophrenia as indexed using available neuroimaging tools before and after use of medication. We will also consider data from pre-clinical studies to provide mechanistic support for our model.

## Prologue

There is substantial evidence, including data from randomized controlled clinical trials, that strongly supports the efficacy of antipsychotics for the acute treatment of psychosis and the prevention of relapse. Specifically, correlational evidence suggests that early intervention, reduced duration of untreated psychosis and early response to treatment might improve longer-term clinical outcomes.^1–3^ However, some concerns have been raised suggesting that treatment with antipsychotic medication might adversely affect long-term outcomes for individuals with a diagnosis of schizophrenia.^1,2^ This includes the risk for extrapyramidal side effects with typical or first-generation antipsychotic drugs, such as haloperidol, and the well-documented adverse metabolic side effects of atypical, or second-generation antipsychotic drugs such as olanzapine, but also clozapine. These include weight gain, elevated risk for metabolic syndrome and potential for cardiovascular toxicity.^4–7^ In addition to these established concerns, we may now add the potential association between antipsychotic drug treatment and a reduction in both brain volume and cortical thickness^8^ as well as dopamine receptor sensitization.^9^ While this has been suggested to potentially make patients vulnerable to relapse and illness progression^10^ this suggestion remains controversial, at least in the context of human studies. Furthermore, it is also hotly debated^11,12^ since disentangling the effects of antipsychotic drug and illness severity is challenging. For example, individuals with greater illness severity may require higher doses of antipsychotics, both of which could be associated with a greater degree of brain adaptations, both functionally and structurally.^12^ Furthermore, volumetric changes after an initial period of antipsychotic drug treatment have been linked to positive drug effects.^13^ On the other hand, some evidence has been found to support a negative long-term effect of maintenance antipsychotic treatment on outcomes, as compared with withholding treatment, although again, this remains a source of debate.^11,14^ Overall, while antipsychotics seem largely effective, strategies for treatment discontinuation or alternative treatment approaches may benefit a subgroup of patients, and further studies are required to clarify any potential noxious effects of drug action and to develop biomarkers that can enable individualized treatment and inform shared decision-making.^11^

## A Metabolic Perspective on The Long-term Effects of Antipsychotic Medication on Clinical Course in Schizophrenia

The purpose of this paper is to propose (1) a model to explain potential malevolent brain metabolic effects of antipsychotic action that may affect a sub-population of patients and (2) a set of biomarkers that could be used to both select the population of interest and test experimentally the hypothesis at hand.

The following sections are organized as follows: “Do Antipsychotic Drugs Have the Potential to Induce Neurodegeneration?” section reviews the evidence supporting the putative association between antipsychotic treatment and neurodegeneration by looking at structural imaging data and their intricate relationship with disease severity and treatment. “The Brain of Individuals With Schizophrenia Exhibits Hypometabolism Before Treatment” and “Antipsychotic Treatment Normalizes Brain Glucose Metabolism” sections bring in foreground the basis of our proposal by recalling that one of the effects of antipsychotic treatment is the “normalization” of glucose metabolism that, at baseline, is reduced in the brain tissue of patients with schizophrenia. “Cerebral Perfusion as a Proxy for Metabolism?” section completes the evidence of the 2 previous sections by looking at matching data from cerebral blood flow imaging studies. “Evidence of Brain Metabolic Distress in Schizophrenia” section notes that the apparent normative effect on metabolism of antipsychotic medication is associated with metabolic distress as evidenced by altered pH and increased lactic acid levels in patients and pre-clinical models. This motivates the focus of “Oxidative Metabolism in Psychosis” section on the available evidence of abnormalities in oxidative metabolism in patients, while “Autophagy in Psychosis” section gathers the available evidence on mitochondrial deficits and autophagy in these subjects. Finally, “Proposal: A Metabolic Cost of Symptom Normalization” section contextualizes and outlines our proposal that the normalization of glucose metabolism resulting from the use of medication may not be sustained by a mirrored increase in oxidative metabolism with ensuing tissue remodeling and stress.

## Do antipsychotic Drugs Have the Potential to Induce Neurodegeneration?

### Clinical Evidence

A large literature has compared first-generation antipsychotics (FGAs) and as well as second-generation antipsychotics (SGAs) on the basis of potential effects on oxidative stress, excitotoxicity, and inflammation, and these findings have generally favored SGAs.^15^ An equally large literature, summarized in table 1, reports on structural neuroimaging data to provide evidence that treatment with FGAs and SGAs may be associated with reduced regional gray matter volume, particularly in the frontal and temporal lobes.

**Table 1.** Collected Evidence on the Association Between Neurodegeneration and Medication in Patients With Schizophrenia

Authors	Type of Study	Results
Moncrieff et al^16^	Meta-analysis, *n* ~ 650	Progressive reduction of brain size and enlargement of brain spaces in people who are taking antipsychotic drug
Navari et al^17^	Meta-analysis, cross-sectional and longitudinal studies	Global volumetric reductions, greater in frontal and temporal lobes, with greater association with typical than with atypical antipsychotics.
Smieskova et al^18^	Meta-analysis, cross-sectional and longitudinal studies	Typical and atypical antipsychotics associated with reduced frontal and temporal lobe volume.
Van Erp et al^19^	Multi-center, *n* ~ 4500	Regional cortical thickness negative correlated with medication, disease severity, and duration
Haijma et al^20^	Meta-analysis, *n* ~ 18 000	Gray matter cortical reductions but subcortical increases associated with longer illness duration and higher dose of medication.
Fusar-Poli et al^21^	Meta-analysis, *n* ~ 800	General gray matter reductions in drug naïve subjects
Fusar-Poli et al^22^	Meta-analysis, *n* ~ 1800, follow-up ~ 72 wk	Gray matter volume decreases associated with cumulative antipsychotic exposure
Huhtaniska et al^23^	Meta-analysis, *n* ~ 700, follow-up > 2 y	Gray matter volume decreases and basal ganglia increases associated with cumulative antipsychotic exposure
Ho et al^24^	Longitudinal, *n* ~ 200, follow-up ~7 y	Gray matter decreases associated with cumulative antipsychotic exposure.
Andreasen et al^25^	Longitudinal, *n* ~ 200, follow-up ~7 y	Decreases of total and regional (eg, frontal) cerebral volume associated with number of relapses and cumulative antipsychotic exposure

Since brain regions associated with gray matter volume loss or cortical thickness reductions during antipsychotic treatment are also reduced in medication-naive patients it is difficult to determine whether brain volume loss reflects progression of illness compared with a medication effect, or an interaction between the two. To try to disentangle the effect of antipsychotics on brain structure from the illness correlates, the role of antipsychotics has also been investigated in healthy volunteers, thus independently of any disease-specific confound. Tost et al^26^ found reduced striatal volume after acute intravenous administration of a high dose of haloperidol (5 mg) in healthy volunteers. However, this finding was not replicated following acute oral administration of 3 mg haloperidol.^27^

### Methodological Caveats

It is worth remembering at this point that a general note of caution should be used in the interpretation of volumetric changes in MRI studies, since MRI is not a direct measure of brain “structure” or “volume” per se. Rather, the measurements obtained from MR images reflect changes in image intensity as a function of tissue water relaxation times (T1, T2), both of which may be influenced by confounding factors such as smoking, alcohol and drug use, body-mass index, corticosteroid levels, exercise regimes and general health^28^ or medication: eg, lithium has been shown to reduce T1 relaxation time in cortical gray matter.^29^ In this context, it is notable that a quantitative assessment of T1 found no effect of acute administration of haloperidol, olanzapine or risperidone in healthy volunteers.^27^ Moreover, in rats chronically administered with haloperidol or olanzapine using clinically comparable dosing and pharmacokinetics, no effects on T2 relaxation time were observed.^30^ Nonetheless, careful use of language when describing the effects of medication on the brain as measured from structural MR images is warranted.

### Pre-clinical Evidence

Controlled studies of antipsychotic drug exposure in animal models have drawn further attention to the potential effect of long-term antipsychotic exposure on brain volumetric data but also to tissue measures. The studies are reported in table 2.

**Table 2.** Collected Evidence on the Effect of Antipsychotics in Pre-clinical Models

Authors	Type of Study	Results
Dorph-Petersen et al^31^	Macaque monkeys, haloperidol/olanzapine for >18 mo.	10% reduction in brain volume.
Konopaske et al^32,33^	Same as above.	Decrease in astroglia
Vernon et al^30^	Rats, haloperidol/olanzapine for 8 wk.	10% reduction in brain volume (but not after 4 wk).
Vernon et al^34^	Replication of study above.	Same brain reductions that were proportional to dose and reversible on drug withdrawal.
Vernon et al^35^	Follow-up of Vernon et al^30^ with voxel-wise morphometry.	Volumetric reductions concentrated in anterior cingulate and parietal cortices due to loss of neuropil but no effect on hippocampus.^36^ Iba1+ ameboid microglia increased.
Bloomfield et al^37^	Rats, haloperidol for 2 wk.	No change in microglia morphology in cingulate cortex
Guma et al^38^	Mice, chronic haloperidol, 9 wk	Pattern of gray matter volume changes in D2 receptor KO mice mimics that seen with chronic antipsychotic exposure. Chronic antipsychotic treatment in D2 receptor KO mice does not lead to additional volume changes.
Guma et al^39^	Same design and study above	Gray matter volume decreases that were reduced in mice lacking D3 receptors

Collectively, these data provide a nexus of causality that chronic exposure to typical or atypical antipsychotic drugs can induce changes in brain volume that are detectable on MRI and reflect genuine tissue changes. At the cellular level, there is, however, no evidence for neuronal loss per se and whilst both astrocyte and microglial cell density and morphology are found to be altered, it is unknown if this represents a homeostatic response or an ongoing, detrimental inflammatory process.^8,40^ Hence, the suggestion that antipsychotic treatment may induce an active, neurodegenerative process is not confirmed by these data. It needs to be pointed out, however, that the aforementioned studies were all done in normal rats, while antipsychotic drugs are given to patients with schizophrenia. Moreover, in many of the rodent studies, the period of antipsychotic drug exposure typically ranges from 4 to 8 weeks. Whilst it is almost impossible to draw accurate comparisons as to how long this is equivalent to in higher species such as primates or man, as a very rough approximation, ~12 days of a rat lifetime may equate to 1 human year.^41^ Based on this assumption, 4 weeks could be equivalent to as much as 2 human years, whilst an 8-week treatment may potentially reflect ~4–5 human years. It is notable then, that although it may be argued rodent studies do not simulate a lifetime of APD treatment, the data from primates, in which the duration of antipsychotic exposure was 2.5 years (table 1), is remarkably similar in terms of direction and magnitude of effect to that seen in rats exposed for only 8 weeks. Nevertheless, very long-term studies in rodents will be necessary to directly address this issue.

Overall, these converging lines of evidence from clinical and pre-clinical studies suggest that there is a potential association between long-term exposure to antipsychotic medication and the potential for loss of gray matter volume. What pre-clinical studies have yet to fully unravel however is the mechanistic nexus between the two. Furthermore, clinical studies have yet to determine the functional and/or clinical relevance of potential medication effects on brain structure in patients with schizophrenia. To address these gaps in our knowledge, we turn to the basis of our proposal and specifically to one of the functional end-points that is demonstrably changed by medication, metabolism; in the following section we review the available data on glucose metabolism in patients with schizophrenia measured first at baseline and, secondly, when metabolic imaging was used longitudinally to observe the effects of treatment.

## The Brain of Individuals With Schizophrenia Exhibits Hypometabolism Before Treatment

Early investigations on glucose metabolism using Positron Emission Tomography (PET) and [18F]-fluorodeoxyglucose (FDG) in unmedicated patients with schizophrenia reported significantly decreased glucose utilization in frontal, striatal and thalamic regions, with the greatest response to antipsychotics observed in those individuals with greatest reductions in glucose utilization in these brain regions.^42,43^ These findings were not generalized (see refs.^44,45^) but have been replicated in numerous subsequent studies (see meta-analysis in ref.^46^ and recent review in ref.^47^) as well as in postmortem tissue and pre-clinical models where insufficient glycolytic activity was most evident in neurons more than astrocytes.^48^ Heterogeneity in the results may indicate varying levels of baseline metabolism in sub-populations^49,50^ but may also be explained by methodology; the early work^42^ that used the more rigorous and logistically cumbersome absolute quantification of glucose metabolic rates has been followed by simplified approaches that necessitated some form of regional count normalization (eg, to injected dose or global/regional brain radioactivity concentrations) that do not inform on metabolic rates and therefore will not be considered here.^51^ The following section will therefore focus only on studies where absolute metabolic measures (eg, gold standard) were reported.

## Antipsychotic Treatment Normalizes Brain Glucose Metabolism

Early work on cohorts of patients responding to treatment with antipsychotics focused on haloperidol treatment that had a “normalizing” effect on glucose metabolism in the striatum and increased metabolism in the cortex, although the anterior-relative-to-posterior gradient remained unchanged. In contrast, patients who did not respond clinically to antipsychotic treatment showed no change in striatal metabolism and a worsening of hypofrontality following haloperidol treatment.^47,52–56^ Later work compared the effects of olanzapine and sertindole to the effects of haloperidol. Buchsbaum et al^55^ reported that, while haloperidol increased striatal metabolism and did not affect frontal metabolism, olanzapine increased metabolism in the frontal lobe more than in the occipital lobe, correcting the hypofrontality seen in FDG-PET studies of schizophrenia. While both medications increased thalamic metabolism, haloperidol increased striatal metabolism more so than olanzapine.

In a 12-week double-blind crossover trial, patients with schizophrenia received sertindole or haloperidol for 6 weeks and then received a FDG-PET scan.^54^ Patients were then crossed over to the other treatment and received a second set of scans at week 12. Patients were also compared with a group of unmedicated patients with schizophrenia and a group of healthy controls. The main finding was that sertindole increased metabolism in the dorsolateral prefrontal cortex and lowered metabolism in orbitofrontal cortex compared to haloperidol.

These data in clinical cohorts are not matched by pre-clinical studies in normal rodents. For example, acute challenges with haloperidol and clozapine generally result in the reduction of cerebral metabolic rates.^57,58^ In contrast, chronic intermittent exposure of rats to the N-methyl-d-aspartate (NMDA) receptor antagonist phencyclidine (PCP) resulted in metabolic reductions within the prefrontal cortex, thalamic reticular nucleus and auditory cortex, which were partially normalized by haloperidol and fully normalized by clozapine; the latter also fully recovered reduced parvalbumin (PV) immunoreactivity, which was reduced by PCP in the above mentioned cortical and sub-cortical areas.^59^ These data further emphasize the importance of examining drug effects in both naïve brains and in a pathological context with relevance to schizophrenia.

## Cerebral Perfusion as a Proxy for Metabolism?

The brain has the highest metabolic requirements of any organ in the body with brain oxygen consumption accounting for 20% of basal oxygen consumption at rest and relies almost completely on oxygen-dependent metabolism of glucose for energy production. This is reflected by the high rate of cerebral blood flow (rCBF) that is continuously adjusted to meet metabolic demand.^60^ Indeed a large number of imaging studies in unmedicated cohorts of individuals with schizophrenia, including discordant monozygotic twins,^61^ have demonstrated abnormalities in brain hemodynamics that are generally consistent with metabolic hypofrontality. Frontal hypoperfusion has been documented in clinical populations involving first episode, antipsychotics-naïve^62–65^ as well as untreated individuals with chronic disease.^66^ Hypoperfusion has also been documented in middle and anterior cingulate, temporal, and parietal regions.^67–69^ However increased rCBF has also been reported in the hippocampus^70^ as well as basal ganglia, and midbrain^71^ of individuals at clinical high risk of psychosis; this follows evidence from pre-clinical models that suggests that hippocampal hyperactivity drives increased activity in the midbrain and basal ganglia in psychosis^72^; importantly, however, in these studies regional rCBF were normalized to global values, mirroring some of the contradictory results obtained in glucose metabolism studies.

The functional effects of antipsychotics measured with rCBF have been largely matching the early metabolic work obtained by PET and FDG. rCBF measures obtained by PET and [15O]H_2_O showed that drug naïve first-episode psychosis patients had increased perfusion after treatment with antipsychotics.^73–75^ Similar results were also obtained in healthy volunteers using a single dose of antipsychotics.^76^ These early PET measurements were then confirmed by later work using Arterial Spin Labelling (ASL), a Magnetic Resonance Imaging (MRI) sequence designed to quantitatively measure regional cerebral blood flow.^27,77–79^

## Evidence of Brain Metabolic Distress in Schizophrenia

There is evidence of increased lactate levels and reduced pH in individuals with schizophrenia, including direct measurements from CSF samples and indirect measurements derived from Magnetic Resonance Spectroscopy (MRS).^80–84^ Importantly a number of postmortem studies have also found decreased pH in the brain tissue of patients with an antemortem diagnosis of schizophrenia. This finding has also received meta-analytic support,^85^ and appears independent from confounding factors (eg, postmortem interval and agonal state).

It has been hypothesized that decreased pH may be related to increased concentration of lactic acid. However, in contrast to the previous notion that an increase in lactic acid represents evidence for primary metabolic abnormalities in schizophrenia, there is evidence that this increase is secondary to prior antipsychotic treatment.^86^ Secondly, there is an excellent correlation between lactate levels in the cerebellum and pH, and that this correlation is particularly strong in tissue from patients with a diagnosis of schizophrenia.

Third, in rats, chronic haloperidol (0.8 mg/kg/day) and clozapine (5 mg/kg/day) exposure for 4 weeks increase lactic acid concentration in the frontal cortex relative to vehicle controls^86^ although this has not been replicated by repeated acute injection of haloperidol (28.5 mg/kg every 3 weeks).^84^ Although further pre-clinical experiments with longer duration of exposure and in the context of illness pathology are needed, existing data suggest that lactate increases in postmortem human brain of patients with schizophrenia could be possibly linked with decreased pH and that these changes are possibly related to antipsychotic treatment rather than a primary metabolic abnormality in the prefrontal cortex of patients with schizophrenia.^86^

## Oxidative Metabolism in Psychosis

The brain is an organ with the highest metabolic cost sequestering between 50% and 65% (depending on age) of the body resting state metabolic requirements with minimal reserve capacity^87^; hence the observation of abnormal lactate and pH levels is a clear indication of metabolic distress and raises the question on the metabolic oxidative capacity of brain tissues in individuals with schizophrenia. Mitochondrial dysfunctions have been proposed to be a key pathway in the pathogenesis of schizophrenia.^88–91^ In fact, autophagy and control of mitochondrial quality is believed to play a significant role in the pathophysiology of this disorder.^92–94^ The ensuing energy is likely to induce the compensatory changes in fatty metabolism observed in this disorder.^95^

### Peripheral Evidence From Clinical Studies

In platelets and lymphocytes of patients with schizophrenia, there is evidence of altered complex I activity, which has likely seriously effects on cellular respiration.^96–99^ In the brain, a parallel transcriptomics, proteomics, and metabolomics study on postmortem tissue has found that approximately half of the altered proteins identified by proteomics in patients with schizophrenia were associated with mitochondrial function and oxidative stress responses and that proteomic alterations were mirrored by transcriptional and metabolite perturbations.^100^

### Imaging Studies of CMRO_2_

The evidence above is contrasted by a remarkable lack of in vivo imaging studies on oxygen metabolism in schizophrenia. Seymour Kety and colleagues^101^ were the first to obtain in vivo determinations of global metabolic rates of oxygen in 22 patients using the Kety-Schmidt NO_2_ method^102^ and did not report differences from normative values. Gordan et al^103^, replicated the experiment in 24 subjects but this time found that *n* = 10 had metabolic values below the normative threshold and that this group was characterized by a disease duration of 4 years or more. The only reported study on cerebral regional metabolic rates of oxygen (CMRO_2_)^104^ using the triple oxygen method and positron emission tomography (PET)^105^ was performed on predominantly never-treated, acute patients with schizophrenia and in matched controls and did not report observable changes. The likely reason for the paucity of PET studies of oxygen metabolism in these cohorts is the cost and logistical burden of the triple oxygen method that requires, on top of the standard radiochemistry and scanning equipment, additional tools that are necessary to manage the very short life of ^15^O.

## Autophagy in Psychosis

In this context of a bioenergetic challenge due to increased oxidative stress and impaired mitochondrial function, it is worth mentioning that neurons (and perhaps glia) compensate by elevating mitophagy, defined as the process by which healthy cells selectively remove damaged or dysfunctional mitochondria that could harm the cell by generating excessive amounts of reactive oxidative species (ROS) and by the release of pro-apoptotic signals such as cytochrome C.^106^ Of note, bioenergetic challenges such as reduced caloric intake due to intermittent fasting, or aerobic exercise, stimulate both mitophagy and mitochondrial biogenesis, allowing neurons to adapt and increase their degree of synaptic plasticity, which may underlie the beneficial effects of caloric restriction and exercise.^106^ In contrast, poor diet and sedentary lifestyle may down-regulate this adaptive response leading to the accumulation of dysfunctional/damaged mitochondria in neurons, reduced synaptic plasticity potential and worsening cognition.^107^ Mitophagy is also increased in the brain tissue of patients with schizophrenia, suggesting an adaptive response to this bioenergetic challenge, which could be plausibly linked to both synaptic pathology and cognitive impairment in this illness.^108,109^ This also raises the question, however, to what extent, if any, antipsychotic treatment contributes to these processes that may be confounded by or interact with patients’ abnormal lifestyle. As already stated, chronic antipsychotic treatment increases brain lactate, but the influence on mitophagy is relatively poorly understood. There is, however, a growing body of evidence that antipsychotic drugs more broadly influence macroautophagy, the process of controlled lysosomal digestion of cellular macromolecules and organelles, of which mitophagy is one specific component.^110^ For example, chronic olanzapine exposure is reported to increase the expression of autophagy markers and autophagic flux both in vitro and in vivo.^110,111^ These data may indicate that olanzapine-triggered autophagy might be a protective, homeostatic response which, if this does not occur sufficiently or autophagy is inhibited, might unmask a potentially neurotoxic action of the drug.^111^ While much more evidence is required to support this assertion (including studies in rodent models with some pathological relevance for schizophrenia as already mentioned above) these data suggest antipsychotic drug treatment may be associated with elevated cellular homeostatic responses associated with bioenergetic challenges, which we expand on in the next section.

## Proposal: A Metabolic Cost of Symptom Normalization?

### The Evidence and an Interpretation

It is clear that the use of FGAs or SGAs is associated with the normalization of behavior and minimization of positive symptoms in responders but also to the return of glucidic metabolic activity towards the normality range. However, there is no evidence that oxidative metabolism is also recovered; on the contrary, the evidence reviewed so far indicates, albeit preliminarily, that long-term use of antipsychotics may be associated, at least in certain individuals, with increased lactic acid tissue content, perhaps suggestive of ongoing metabolic dysfunction. It might be also possible that in the context of a shifted mitochondrial redox state and neuronal high-frequency firing, lactate might function as an alternative substrate,^112,113^ altering the physiological astrocyte-neuron lactate shuttle and neuronal activity.^114,115^

Hence effective antipsychotic treatment may depend on several pre-conditions that include (but are not limited to) (1) efficient mitochondrial function, (2) adequate regional blood supply, (3) fully functional neurovascular coupling capable of providing adequate amounts of nitric oxide and other vaso-active molecules to sustain the necessary increases on oxygen availability, and (4) sufficient quantities of hemoglobin to carry the additional oxygen demand.

**Fig. 1.**
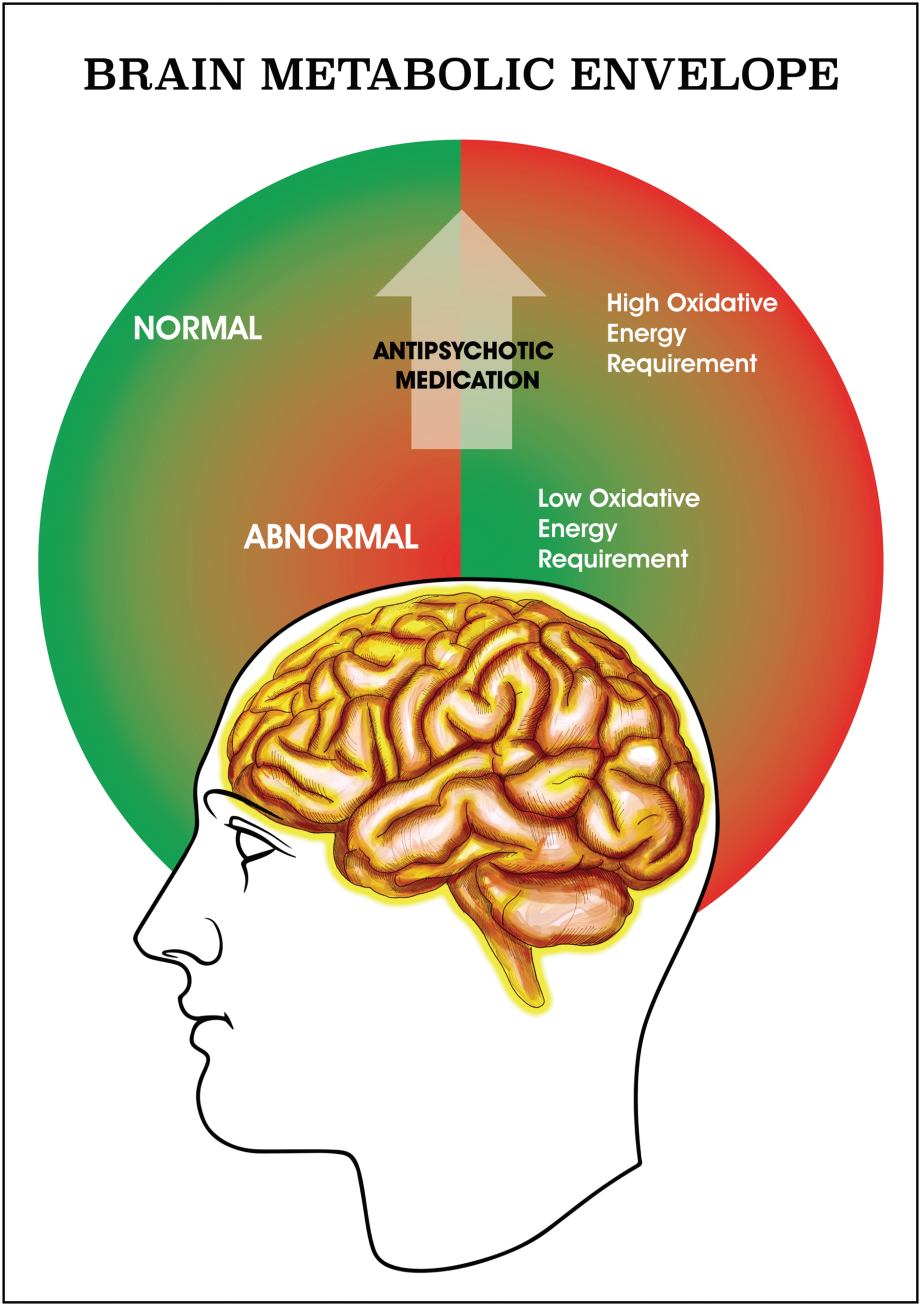
Our model proposes that the brain tissue of patients with schizophrenia is in a homeostatic metabolic state characterized by low metabolism, both glucidic and oxidative. We then propose that antipsychotic medication normalizes behavior and consequently brings back glucose metabolism to normal levels; however, this “normal” high metabolic state may not be matched by the oxidative capacity of a subset of these patients with ensuing detrimental long-term effects and tissue remodeling.

However, if the patients do not have the physiological characteristics to meet these 4 requirements, one can foresee several scenarios in which antipsychotic administration would carry little therapeutic benefit and patients could develop detrimental long-term side effects associated with the inability of the tissue to meet the metabolic demands brought about by the drug (figure 1). It is plausible under these circumstances that this could result in a degree of cellular remodeling and hence, macrostructural changes detectable by MRI, but this needs to be investigated experimentally in both human and pre-clinical models.

### Future Work: Diagnostics

The proposal above is then consistent with a pressing need to personalize treatment and suggests future work to quantitatively and non-invasively assess a number of regional cerebral physiological factors that may predict both treatment efficacy and the likelihood of detrimental side effects (eg, tardive dyskinesia, cognitive impairment, secondary negative symptoms) that have huge impact on patients’ quality of life. Some of these might include: determination of regional CBF using techniques such as Arterial Spin Labelling (ASL),^116^ acquisition of baseline Oxygen Extraction Fraction maps,^117^ pH imaging using amid proton contrast MRI,^118^ imaging brain lactate either through MRI spectroscopy^119^ or MRI and chemical exchange saturation transfer (MRI-CEST).^120^ An interesting addition might be to generate maps of vascular reactivity using CO_2_ administration^121^ to assess the tissue potential for increases in local CBF. All the above could likely be focused on those individuals who exhibit deficits in mitochondrial activity in the periphery measured using either mRNA^122^ or enzymatic assays in blood monocytes^96,123^ or platelets^122,124^ or both; this would obviously assume an association between peripheral and CNS oxidative capacity. Alternatively, one could either induce or trans-differentiate neurons from individual subjects to directly measure their mitochondrial function.^125^ Such studies are necessary to also address the fundamental questions that remain around the potential impact of antipsychotic medication on brain structure and function in patients with schizophrenia and determine the clinical relevance, if any, of these changes, in terms of either symptom remission or treatment non-response, long-term side effects and cognitive impairment.

### Future Work: Complementary Medication

If the model proposed in “The Evidence and an Interpretation” section is correct, then the need for diagnostics will be mirrored by the need of further considerations on long-term maintenance treatments (eg, strategies of treatment discontinuation) and adjunctive therapies to support those who will demonstrate deficits in oxidative metabolism. Presently, treatments for patients with mitochondrial disorders are largely restricted to exercise, dietary supplements with some benefit^126^; eg, CoQ10 supplements might be beneficial for treating conditions such as congestive heart failure and Parkinson’s disease. CoQ10 is considered safe and tolerable.^127^ However, in the last few years, new therapies have gone into development and are entering human clinical trials that are active on mitochondrial biogenesis, mitophagy, hypoxia, and mitochondrial dynamics, or bypass biochemical defects and enable mitochondrial replacement therapy.^128^ For example, methylene-blue has been described as a pharmacological treatment that increases mitochondrial respiration resulting in neuroprotective and cognitive-enhancing effects in both clinical and pre-clinical studies in Alzheimer’s disease.^129–131^ Hence future experimental interventions could be aligned to these therapeutic developments and tailored to ameliorate brain metabolic function in these patients.

A second but important line of investigation should also target the direct effects of antipsychotics on metabolism; it is known that first-generation antipsychotics cause a certain degree of metabolic disorders while second-generation APDs, particularly clozapine and olanzapine, have worse metabolic side-effects that include, obesity, hyperlipidemia, insulin resistance, hyperglycemia and diabetes^132,133^; the mechanisms underlying these comorbidities still need to be clarified.^134^ However, there is recent albeit preliminary evidence that antipsychotics may have direct effects on mitochondrial function, with one study reporting a direct association between antipsychotic treatment and mitochondrial DNA damage^135^; if this were proven to be the case then the scientific program outlined here could obviously be of great value to tune the risk/benefit of current therapeutic strategies while more advanced new therapies are under development.
