# Corrigendum to: Normalizing the Abnormal: Do Antipsychotic Drugs Push the Cortex Into an Unsustainable Metabolic Envelope?

**DOI:** 10.1093/schbul/sbz139

**Published:** 2020-01-17

**Authors:** Federico E Turkheimer, Pierluigi Selvaggi, Mitul A Mehta, Mattia Veronese, Fernando Zelaya, Paola Dazzan, Anthony C Vernon

In the original publication of this article, the terms (FGAs) and (SGAs) in the section “Do antipsychotic Drugs Have the Potential to Induce Neurodegeneration?” were only partially defined. This text has since been corrected. The authors regret this error.

